# MicroRNA characterization in equine induced pluripotent stem cells

**DOI:** 10.1371/journal.pone.0207074

**Published:** 2018-12-03

**Authors:** Lucia Natalia Moro, Guadalupe Amin, Veronica Furmento, Ariel Waisman, Ximena Garate, Gabriel Neiman, Alejandro La Greca, Natalia Lucia Santín Velazque, Carlos Luzzani, Gustavo E. Sevlever, Gabriel Vichera, Santiago Gabriel Miriuka

**Affiliations:** 1 LIAN-CONICET, Fundación FLENI, Buenos Aires, Argentina; 2 Kheiron Biotech, Pilar, Buenos Aires, Argentina; Universidade de São Paulo, BRAZIL

## Abstract

Cell reprogramming has been well described in mouse and human cells. The expression of specific microRNAs has demonstrated to be essential for pluripotent maintenance and cell differentiation, but not much information is available in domestic species. We aim to generate horse iPSCs, characterize them and evaluate the expression of different microRNAs (miR-302a,b,c,d, miR-205, miR-145, miR-9, miR-96, miR-125b and miR-296). Two equine iPSC lines (L2 and L3) were characterized after the reprogramming of equine fibroblasts with the four human Yamanaka‘s factors (*OCT-4/SOX-2/c-MYC/KLF4*). The pluripotency of both lines was assessed by phosphatase alkaline activity, expression of *OCT-4*, *NANOG* and *REX1* by RT-PCR, and by immunofluorescence of OCT-4, SOX-2 and c-MYC. *In vitro* differentiation to embryo bodies (EBs) showed the capacity of the iPSCs to differentiate into ectodermal, endodermal and mesodermal phenotypes. MicroRNA analyses resulted in higher expression of the miR-302 family, miR-9 and miR-96 in L2 and L3 *vs*. fibroblasts (p<0.05), as previously shown in human pluripotent cells. Moreover, downregulation of miR-145 and miR-205 was observed. After differentiation to EBs, higher expression of miR-96 was observed in the EBs respect to the iPSCs, and also the expression of miR-205 was induced but only in the EB-L2. In addition, *in silico* alignments of the equine microRNAs with mRNA targets suggested the ability of miR-302 family to regulate cell cycle and epithelial mesenchymal transition genes, miR-9 and miR-96 to regulate neural determinant genes and miR-145 to regulate pluripotent genes, similarly as in humans. In conclusion, we could obtain equine iPSCs, characterize them and determine for the first time the expression level of microRNAs in equine pluripotent cells.

## Introduction

Generating domestic animal embryonic stem cells has been proved to be difficult and has sparked the interest on induced pluripotent stem cells (iPSCs) in these species [1]. In the horse, this technology has emerged as a promising therapeutic alternative for musculoskeletal injuries in athletic animals. In contrast to mesenchymal stem cells that have been used for tissue regeneration in the last decade [2–4], iPSCs have the advantages of indefinite proliferation and higher differentiation potential [5]. In fact, equine iPSCs myogenic differentiation capability has already been probed for skeletal muscle regeneration [6].

Several groups have reported the generation of iPSCs in the horse by reprogramming adult fibroblasts, fetal fibroblasts, keratinocytes and adipose-derived stem cells [6–9]. With these reports, it is clear that equine iPSCs can be generated from different sources, with the capacity to differentiate into all three germinal layers both *in vitro* and *in vivo*. However, there is scarce information about the processes involved in equine cell reprogramming, stem maintenance and cell differentiation, in contrast to other species like human or mouse. It is well known from these two models that the biogenesis of microRNAs is a critical process for cell reprogramming and differentiation [10–14]. microRNAs are short non-coding RNA molecules that mainly regulate mRNAs by post-transcriptional targeting of the 3’UTR tail [15], either bloking their translation or inducing its degradation [16]. However, interaction of microRNAs with other regions, such as the 5′ UTR, coding sequences, and gene promoters, have also been reported [17, 18]. It has been demonstrated that a single microRNA can regulate the expression of hundreds of mRNA targets [19], a property given by a short sequence (called ‘seed’) in position 2-8 from the 5’ end. The seed is essential for the binding of the microRNA to the mRNA and complementary to microRNA-recognition sequences present in the 3’UTR tail of the mRNAs [20]. microRNA regulation capacity is so marked that human and murine iPSCs have been efficiently generated by only inducing the expression of the miR-302/367 cluster, without the need of the *OCT-4/SOX-2/KLF4/c-MYC* reprogramming factors [21, 22]. The mir-302/367 cluster is a central player of pluripotent stem cell maintenance, self-renewal and differentiation [23, 24]. On the other hand, other microRNAs are involved in lineage determination by repressing the expression of *OCT-4*, *SOX-2* and *KLF4*, such as miR-145 that is involved in mesoderm and ectoderm differentiation [25, 26]. miR-9 and miR-96 were shown to be involved in neurogenesis [27–29], and miR-125b was shown as an important regulator of stem cells differentiation to mesoderm and cardiac muscle [30], among others. All together, microRNAs has been extensively shown to regulate critical processes in embryo development.

Until now, there is no information about equine microRNA expression neither in iPSCs nor in embryos. The possibility of elucidating the microRNA expression and regulation in equine iPSCs could enable us to manipulate them and induce cell reprogramming and differentiation more efficiently in this species as was previously demonstrated in the mouse [31]. The objective of this work is to generate horse iPSCs lines, to characterize them and to evaluate the expression of specific microRNAs critical for embryo development. We are interested in exploring whether these processes are similar to those reported for the human and the mouse that have been extensively studied.

## Materials and methods

### Reagents

Except otherwise indicated, all chemicals were obtained from Thermo Fisher Scientific (Waltham, Massachusetts, USA). Media were prepared weekly and filtered through 0.22 *μ*m membranes.

### Animal care and use of research animals

The present study was carried out following the Guide for the Care and Use of Agricultural Animals in Agricultural Research and Teaching. The protocols involving animal manipulations were approved by the Institutional Committee for the Care and Use of Experimental Animals of the San Martin National University, Buenos Aires, Argentina (CICUAE-UNSAM, Permit Number: 001/16).

### Cell culture

Equine fibroblasts were obtained from the skin sample of a spontaneously aborted 5-month-old foetus of Polo Argentino breed. Cells were cultured in Dulbecco´s modified Eagle´s medium (DMEM, catalog number 12100-46) supplemented with 10% foetal bovine serum (FBS) and 1% penicillin/streptomycin (Pen/Strep, 15140-122) in a 5% CO_2_, humidified atmosphere at 37°C. After reprogramming, the iPSCs were cultured over inactivated mouse embryonic fibroblast (iMEFs) in eqHES medium [DMEM/F12 (11320033) with 20% Knock-out serum replacement (A3181502), 10 ng/ml basic fibroblast growth (PHG0263), 1% Pen/Strep and 10 ng/ml Human Leukemia Inhibitory Factor (hLIF) (PHC9484)].

### Lentiviral vector production

Cell reprogramming was performed by using the EF1a-hSTEMCCA-loxP (STEMCCA) lentiviral cassette containing the human genes *OCT-4/SOX-2/KLF4/c-MYC*, generously provided by Dr. Gustavo Mostoslavsky [32]. The lentiviral vectors were produced as previously described by our lab [33], with minor modifications. Briefly, HEK-293 T cells were transfected with the FuGENE6 containing the STEMCCA and helper plasmids. The supernatants were collected 72 h post-transfection and ultracentrifuged at 76221 g for 1 h 30 minutes in a SW 40 Ti rotor. The concentrated virus was then resuspended in cold PBS, aliquoted and stored at -80°C until used. Viral titer was determined as previously described by our lab [33].

### Equine iPSCs lines generation by STEMCCA virus

Equine fibroblasts were thawed and cultured as described above for 7 days before infection. On day -1, 1x10^5^ cells were plated on 1 well of a 6 well plate previously coated with 0.1% bovine gelatin. The day of infection (Day 0), cells were incubated for 16 h with the STEMCCA lentivirus at a titer of MOI 1 and 10 μg/ml of polybrene (TR-1003, Sigma Aldrich, San Luis, Missouri, USA). Spinfection was performed by centrifuging the plate at 700 g for 55 minutes before incubation. On day 1, the virus was removed and the cells were cultured in DMEM medium for 5 days when the first colonies appeared. On day 6, the infected cells were trypsinized and plated in different dilutions (1:4, 1:16, 1:40) over iMEFs in eqHES medium to allow colonies to grow. Twelve colonies were selected according to morphology and mechanically picked from the plate after 2 weeks and cultured in the same conditions. Clonal expansion was performed for 2 colonies, which were amplified and characterized (L2 and L3 lines). For each passage, colonies were detached with collagenase (17100017) centrifuged and plated on iMEF in eqHES medium. Both L2 and L3 lines were maintained in culture over 25 passages.

### Alkaline phosphatase staining

Alkaline phosphatase activity was determined in both iPSC lines and fibroblasts using a commercial kit (86R-1KT, Sigma-Aldrich) following the manufacturer’s instructions.

### *In vitro* differentiation to embryo bodies

Differentiation of L2 and L3 to embryo bodies (EBs) was performed as previously described by Breton et al. (2013) [9] with minor modifications. Briefly, L2 and L3 colonies were treated with collagenase to allow detachment, passaged in a non-adherent culture dish and cultured in suspension for 7 days in DMEM with 10% FBS. Under these conditions, EBs were formed and after 7 days they were passaged to gelatin-coated adherent-dishes with the same culture conditions for 2 more weeks. After this time, samples were prepared using TRIzol reagent (15596026) for total RNA extraction or the cells were fixed for immunostaining, as explained bellow.

### RNA extraction, cDNA synthesis and real time PCR

RNA extraction from the different cell lines was performed with TRIzol reagent. For cDNA synthesis, 500-1000 ng of the total RNA was retro-transcribed with MMLV reverse transcriptase (Promega, WI, USA), according to manufacturer’s instructions. For microRNA reverse transcription, cDNA was generated as previously described using SuperScript™ II Reverse Transcriptase (18080044) [34]. For real-time PCR, cDNA samples were diluted 5-folds and PCR was performed with StepOne Plus Real Time PCR System (PE Applied Biosystems, CA, USA). The Fast SYBR Green Master Mix (4385612) was used for all reactions. The housekeeping gene used was RPL7 when mRNAs were analyzed and RNU6B when microRNAs were analyzed. A list of primers is shown in S1 Table.

### Immunostaining

iPSCs lines, EBs, fibroblast cells and horse embryos were fixed in 4% paraformaldehyde for 30 minutes at room temperature, washed with PBS, and then permeabilized for 1 h in a solution containing 0.1% bovine serum albumin/PBS, 10% FBS and 0.1% Triton. After permeabilization, blocking was assessed by 1 h incubation with 3% FBS and 0.1% Tween-20 (Promega, H5152), followed by primary antibody incubation for 2 hours at room temperature. They were then washed and incubated with the secondary antibody and DAPI for 1 h at RT in the dark. Negative controls were performed using only the secondary antibody. Specific antibodies and dilutions are detailed in S2 Table.

### Somatic cell nuclear transfer

Somatic cell nuclear transfer and embryo transfer were performed as previously described by Olivera et al [35, 36]. Briefly, equine oocytes were in vitro matured and enucleated by micromanipulation. After oocyte enucleation, fusion of one L3 iPSC or one original fibroblast cell was applied. The reconstructed embryos were then chemically activated and *in vitro* cultured for one week in order to achieve the blastocyst stage. On day 2 and day 7 cleavage and blastocyst rates were assessed, respectively. Those embryos that reached the blastocyst stage were transferred to recipient mares to continue gestation until birth. Mare synchronization, embryo transfer, gestation monitoring and neonatal care was performed as previously reported by Olivera et al. [35, 36].

### *In-silico* microRNAs target determinations

First we compared the sequences of 10 microRNAs in the horse and the human (miR-302a, miR-302b, miR-302c, miR-302d, miR-205, miR-145, miR-9, miR-96, miR-125b and miR-296), which were obtained in the miRBase database (www.mirbase.com) (Table 1). In order to determine whether the eca-microRNAs could target the same mRNAs as the ones reported in humans and mice, we evaluated the complementarity of the seed sequence of the miR-302 family, miR-96, miR-9 and miR-145 with the 3‘UTR of possible target genes. In most reports the microRNA seed sequence is essential for the binding of the microRNA to the mRNA and it comprises a contiguous string of at least 6 nucleotides beginning at position 2 of the microRNA (the sufficient minimal set of microRNA seed type) [20]. To achieve this, the horse mRNA sequences were obtained from the NCBI-nucleotide database (www.ncbi.nlm.nih.gov). The accession numbers of the genes were: *eqOCT-4* (XM_001490108.6), *eqKLF4* (XM _005605684.2), *eqSOX2* (FJ356148.1), *eqCDK2* (XM_001504790.6), *eqCYCLIN D1* (XM_023654619.1), *eqRHOC* (XM_001917242.5), *eqE2F1* (XM_023626374.1), *eqHES1* (XM_001498844.5) and *eqPAX6* (XM_023646562.1). Sequence alignments were performed with Geneious software and RNA22 software [37].

**Table 1:** microRNA sequences used in this study.

microRNA	Equine Sequences*	Human Sequences*
*mir*—302*a*—3*p*	UAAGUGCUUCCAUGUUUU***A***GUGA	UAAGUGCUUCCAUGUUUU***G***GUGA
*mir*—302*b*—3*p*	UAAGUGCUUCCAUGUUUUAGUAG	UAAGUGCUUCCAUGUUUUAGUAG
*mir*—302*c*—3*p*	UAAGUGCUUCCAUGUUUCAGUGG	UAAGUGCUUCCAUGUUUCAGUGG
*mir*—302*d*—3*p*	UAAGUGCUUCCAUGUUU***U***AGUGU	UAAGUGCUUCCAUGUUU***G***AGUGU
*mir*—205—5*p*	UCCUUCAUUCCACCGGAGUCUG	UCCUUCAUUCCACCGGAGUCUG
*mir*—145—5*p*	GUCCAGUUUUCCCAGGAAUCCCU	GUCCAGUUUUCCCAGGAAUCCCU
*mir*—9—5*p*	UCUUUGGUUAUCUAGCUGUAUGA	UCUUUGGUUAUCUAGCUGUAUGA
*mir*—96—5*p*	UUUGGCACUAGCACAUUUUUGCU	UUUGGCACUAGCACAUUUUUGCU
*mir*—125*b*—5*p*	UCCCUGAGACCCUAACUUGUGA	UCCCUGAGACCCUAACUUGUGA
*mir*—296—5*p*	GAGGGUUGGGUGGAGGCU***U***UCC	GAGGGUUGGGUGGAGGCU***C***UCC

* Underlined letters remark differences between equine and human microRNA sequences.

### Transfection of episomal reprogramming vectors

Equine fibroblasts were transfected with different combinations of episomal reprogramming plasmids that have demonstrated to reprogram human cells [38–40]: pCXLE-hOCT3/4-shp53-F (addgene N°27077), pCXLE-hSK (addgene N°27078), pCXLE-hUL (addgene N°27080), pEP4 E02S CK2M EN2L (addgene N°20924) and pCXWB-EBNA1 (addgene N°37624). Either the 100 *μ*l tip NEON system or FuGENE6 transfection reagent (Roche, 1814443) was used for the transfection of the plasmids, following the manufacturer´s instructions in both cases. The equine fibroblasts were transfected with different conditions of plasmid concentrations, plasmid combinations and NEON settings, which are detailed in S3 Table. All the conditions were evaluated twice and the EGFP-N1 plasmid was used as control of transfection in each procedure. Once transfected, the cells were cultured in gelatin coated p100 in DMEM 10% SFB medium for 5 days. After that time, they were trypsinized and plated in different dilutions over iMEFs in eqHES medium.

### Statistical analysis

Real time PCRs were analyzed with the LinReg PCR software. Statistical differences were analyzed using either paired Student‘s t test or ANOVA. Comparisons between means were assessed using the Tukey test. Statistical analysis for the results of *in vitro* embryo development was performed using non parametric Fisher’s exact test (p<0.05).

## Results

### Fibroblast reprogramming and pluripotency characterization of horse iPSCs obtained by STEMCCA transduction

**Fig 1:**
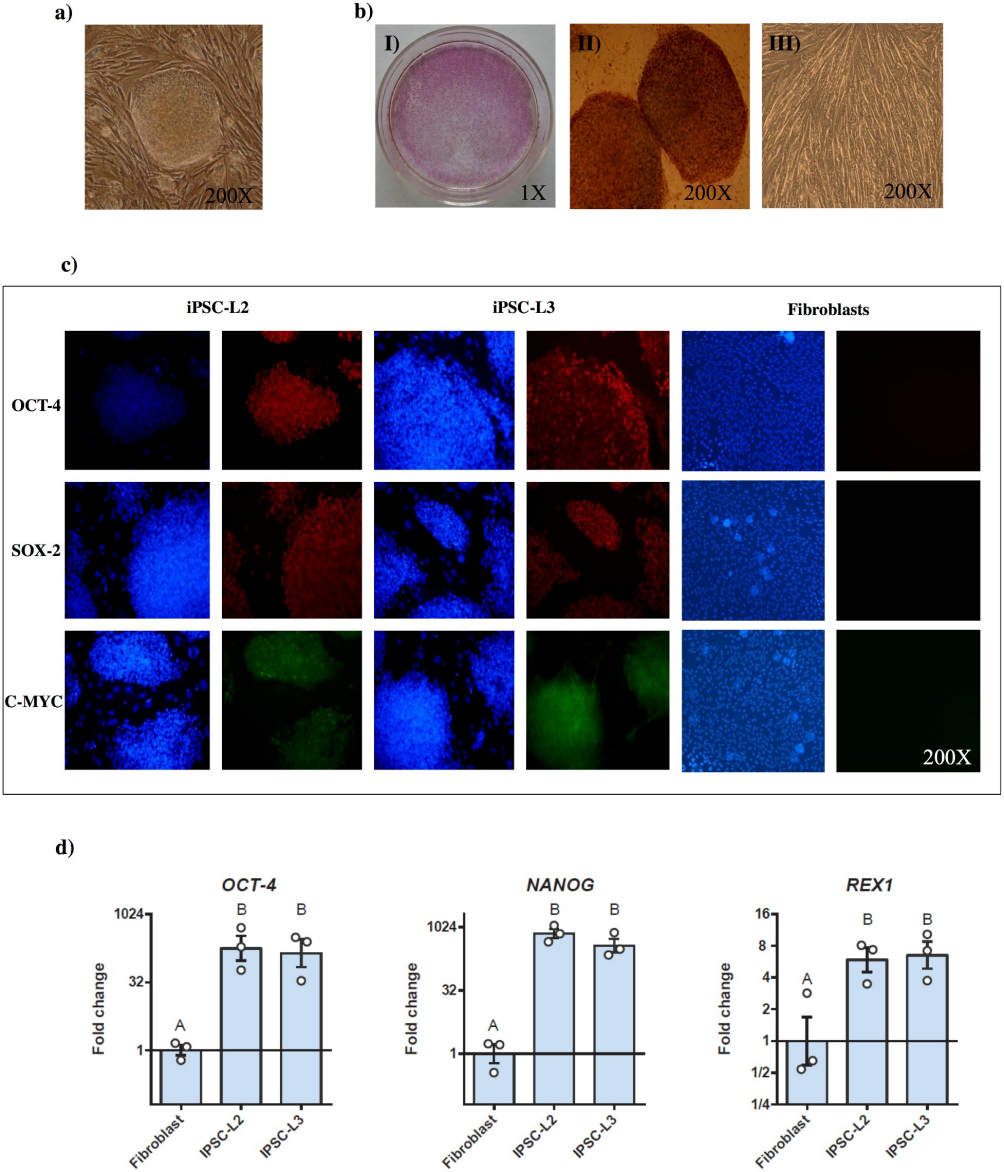
Pluripotent characterization of two horse iPSC lines, L2 and L3. a) A representative colony of equine iPSCs 2 weeks after reprogramming. b) Alkaline phosphatase activity analysis of the L2 iPSC line, I) A 60 mm petri dish full of iPSC colonies observed in pink; II) Two iPSC colonies with high alkaline phosphatase activity; III) Horse fibroblasts negative for alkaline phosphatase activity. c) Inmunostaining of both iPSC lines and horse fibroblasts with the pluripotent markers OCT-4, SOX-2 and c-MYC. In blue nucleous are stained with DAPI d) RT-qPCR analysis comparing the expression of *OCT-4*, *NANOG* and *REX-1* among the original fibroblasts and the L2 and L3 iPSC lines. Results are presented as means ± SEM (n = 3). Data were relativized to fibroblasts. Different letters indicate significant differences (p<0.05).

**Fig 2:**
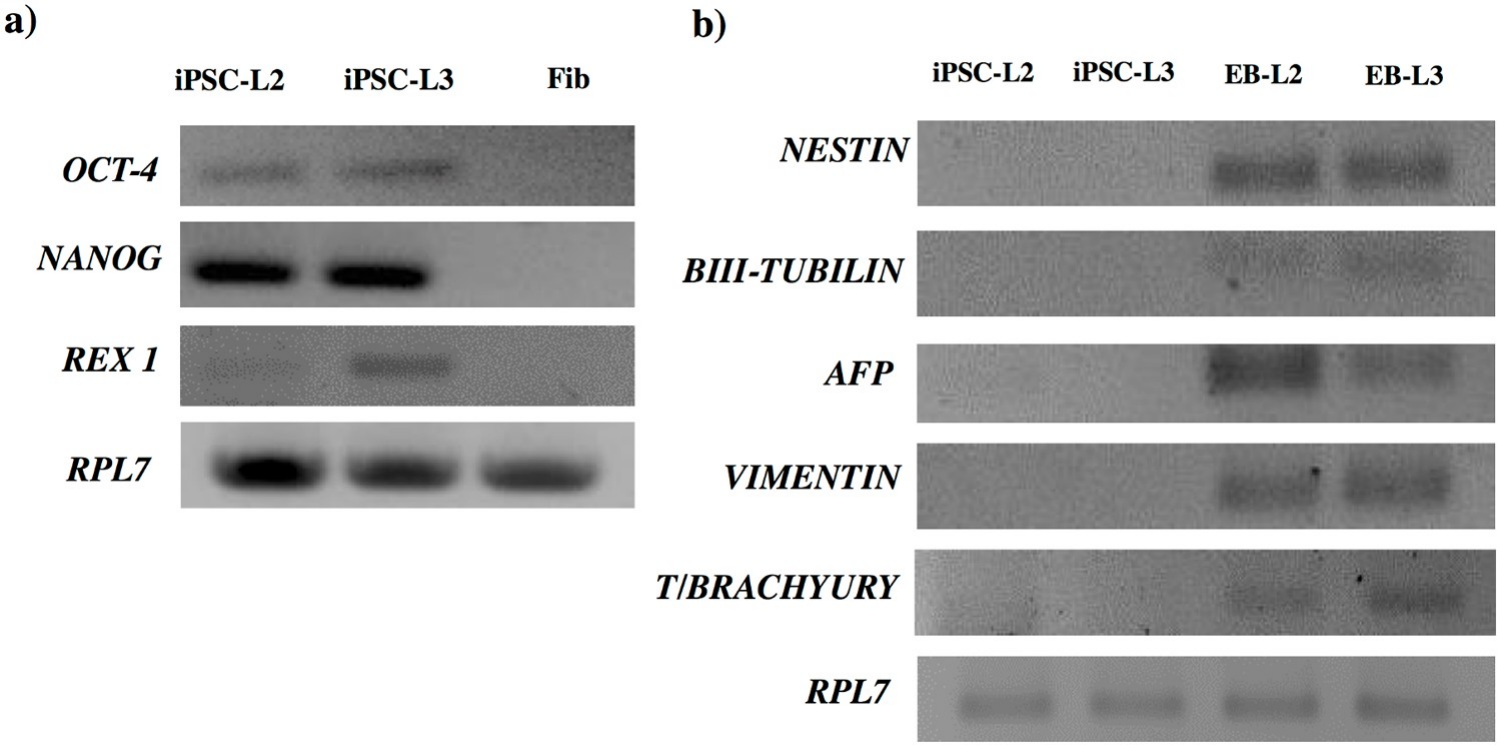
End point PCR analysis of iPSCs. a) the pluripotent genes *OCT-4*, *NANOG* and *REX1* in fibroblasts (Fib) and both iPSCs lines L2 and L3; and b) representative genes from the three germ layers in L2 and L3 iPSCs, and embryo bodies (EB), derived from these both iPSCs lines (EB-L2 and EB-L3, respectively). *RPL7* was used as the housekeeping gene.

We attempted several times to reprogram equine fibroblasts by using different combination of episomal vectors (S3 Table). However, we could not develop equine iPSCs by this strategy. Few reports have demonstrated the capacity to reprogram human cells with episomal vectors [38–40], nevertheless, the equine cells that were transfected with these plasmids showed changes only in shape morphology and overgrowing correlated with partial reprogramming. In contrast, after 10-12 days of viral infection with the STEMCCA lentivirus, small reprogrammed colonies appeared, resulting in horse reprogrammed cells. Several colonies were obtained, and we selected two of them based on the morphology (named L2 and L3) for full characterization (Fig 1a). The pluripotency of both iPSCs lines was assessed by different techniques. We observed high alkaline phosphatase activity in L2 and L3 lines, which has demonstrated to be an indicator of pluripotency, and we did not observe alkaline phosphatase staining in fibroblasts, as expected (Fig 1b). Moreover, high expression of pluripotency markers such as *OCT-4*, *NANOG* and *REX1* was observed by end-point PCR and real time PCR using horse specific primers (Figs 1 and 2). Lower expression of these markers was observed for the fibroblast group by real time PRC and no amplification was detected by end-point PCR in this group (Figs 1 and 2). Moreover, both L2 and L3 iPSCs lines were also positive for expression of OCT-4, SOX-2 and c-MYC proteins, assessed by immunofluorecence staining, not observing any positive signal in horse fibroblasts (Fig 1c). As these three genes are also encoded by the STEMCCA virus, we ensured that the antibodies used can recognize the equine OCT-4, SOX-2 and c-MYC proteins by evaluating them in equine embryos by immunostaining shown in S1 Fig. With this control we confirmed that the antibodies can be used in the horse.

### *In vitro* differentiation

**Fig 3:**
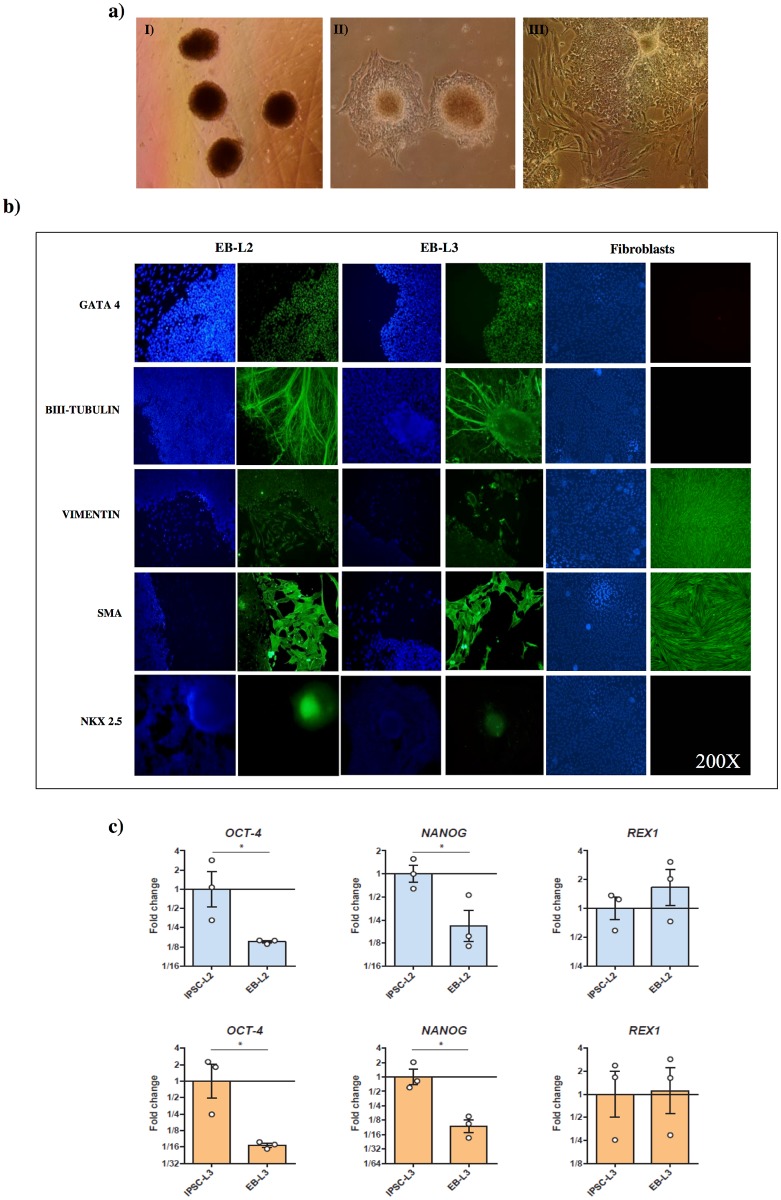
*In vitro* differentiation of L2 and L3 iPSCs to embryo bodies (EB). a) Generation of EBs, I) EBs in suspension for 1 week in DMEM medium, II) EBs after 3 days in adherent dishes, III) EBs after 2 weeks in adherent dishes; b) Inmmunofluorescence of endodermal, ectodermal and mesodermal markers in EBs derived from L2 (EB-L2), L3 (EB-L3) iPSCs lines and fibroblasts as controls. In blue nucleous are stained with DAPI c) RT-qPCR analysis of pluripotent markers in L2 *vs*. EB-L2 and L3 *vs*. EB-L3. Results are presented as means ± SEM (n = 3). Data were relativized to L2 or L3 for EB-L2 and EB-L3, respectively. *Statistically different (p<0.05).

We generated *in vitro* EBs in order to evaluate the capacity of both iPSCs lines to differentiate into the three germ layers. After colony detachment, EBs were formed in few hours in both iPSCs lines (Fig 3a). Both EB-L2 (EBs derived from L2) and EB-L3 (EBs derived from L3) showed expression of the ectodermal markers nestin, *β*III-tubulin, the endodermal marker AFP and the mesodermal markers vimentin and brachyury, by end point PCR. In contrast, non-differentiated iPSCs cells were negative for these markers (Fig 2b). Immunofluorescent staining was also used to determine the protein expression of differentiated cells to the three germ layers. We assessed the expression of GATA-4, *β*III-TUBULIN, VIMENTIN, SMOOTH-MUSCLE ACTIN (SMA) and NKX-2.5 by immunofluorescence in both EB-L2 and EB-L3 and observed a strong specific staining (Fig 3b). As expected, fibroblasts were negative for GATA-4, *β*III-TUBULIN and NKX-2.5, but positive for vimentin and SMA, both mesodermal markers. In addition, *OCT-4* and *NANOG*, but not *REX1*, were down-regulated in the EBs respect to the iPSCs lines (Fig 3c).

### Somatic cell nuclear transfer

We compared the capacity of the horse iPSCs and fibroblasts to generate *in vitro* embryos and viable foals. Results using L3-iPSCs as nuclear donors are shown in Table 2. In both cases, we obtained similar cleavage rates between groups (51/56 (91.1%) and 205/246 (83.3%) for L3-iPSC and fibroblasts, respectively), but no blastocysts were generated with the pluripotent cells as nuclear donors. Those blastocysts generated with the original fibroblasts were transferred to recipient mares and 2 healthy foals were born. With these results we could determine the good quality of the embryos generated.

**Table 2:** Equine cloning using L3-iPSCs as nuclear donors.

Groups	Embryos	Cleavage	Blastocysts	Embryo transfers	Pregnancies	Offspring (%)
*iPSC*—*L*3	56	51 (91.1)	0^a^	-	-	-
*Fibroblasts*	246	205 (83.3)	22 (8.9)^b^	11	2 (18.2)	2 (100)

^(a, b)^ Values with different superscripts in a column are significantly different (Fisher’s exact test p<0.05)

### microRNAs expression in horse iPSCs and differentiated EBs

**Fig 4:**
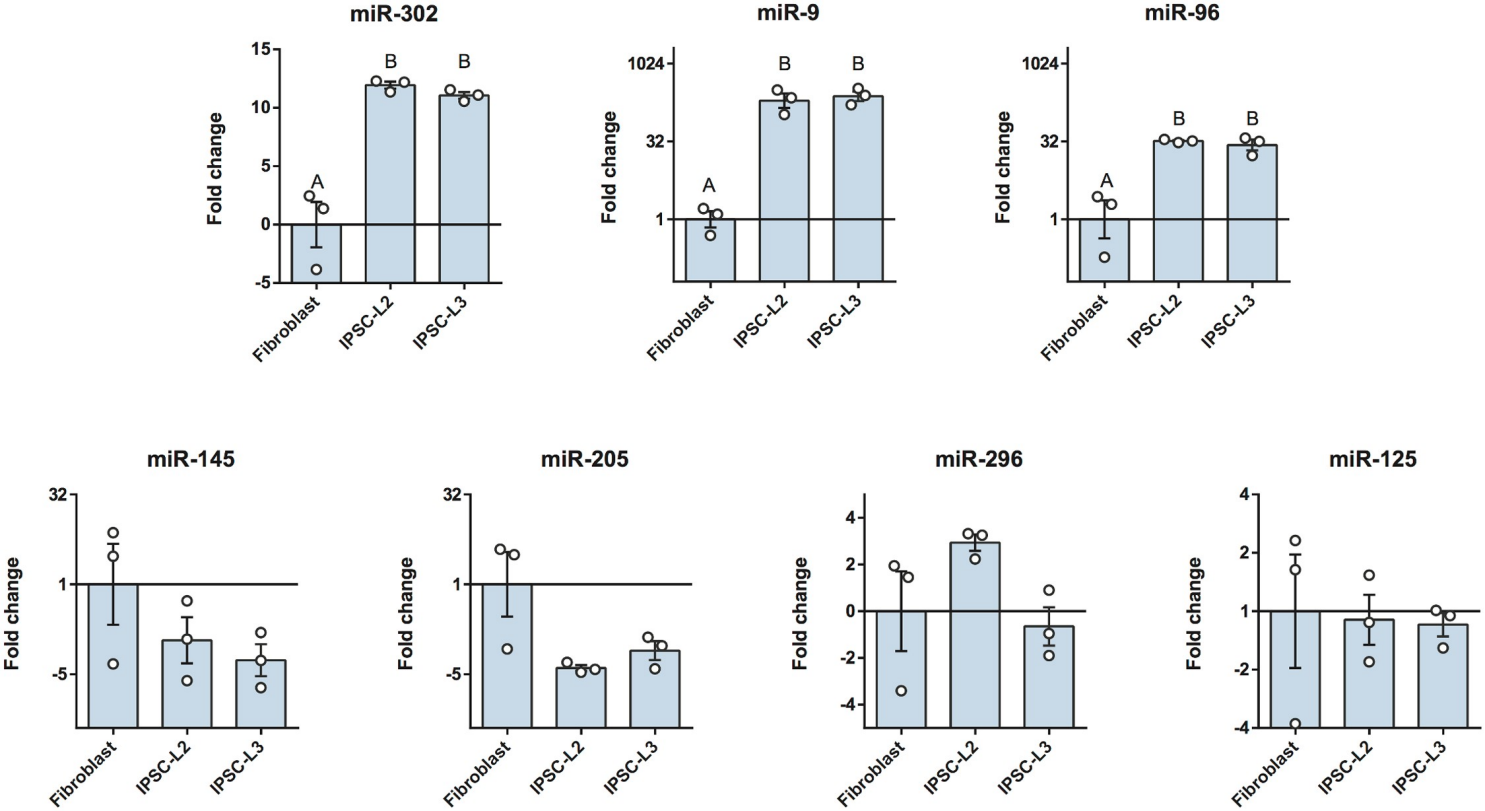
RT-qPCR analysis of 7 microRNAs in the L2 and L3 iPSCs lines and the original fibroblasts. RT-qPCR analysis of 7 microRNAs in the L2 and L3 iPSCs lines and the original fibroblasts. Results are presented as means ± SEM (n = 3). Data were relativized to fibroblasts. Different letters indicate significant differences (p<0.05).

**Fig 5:**
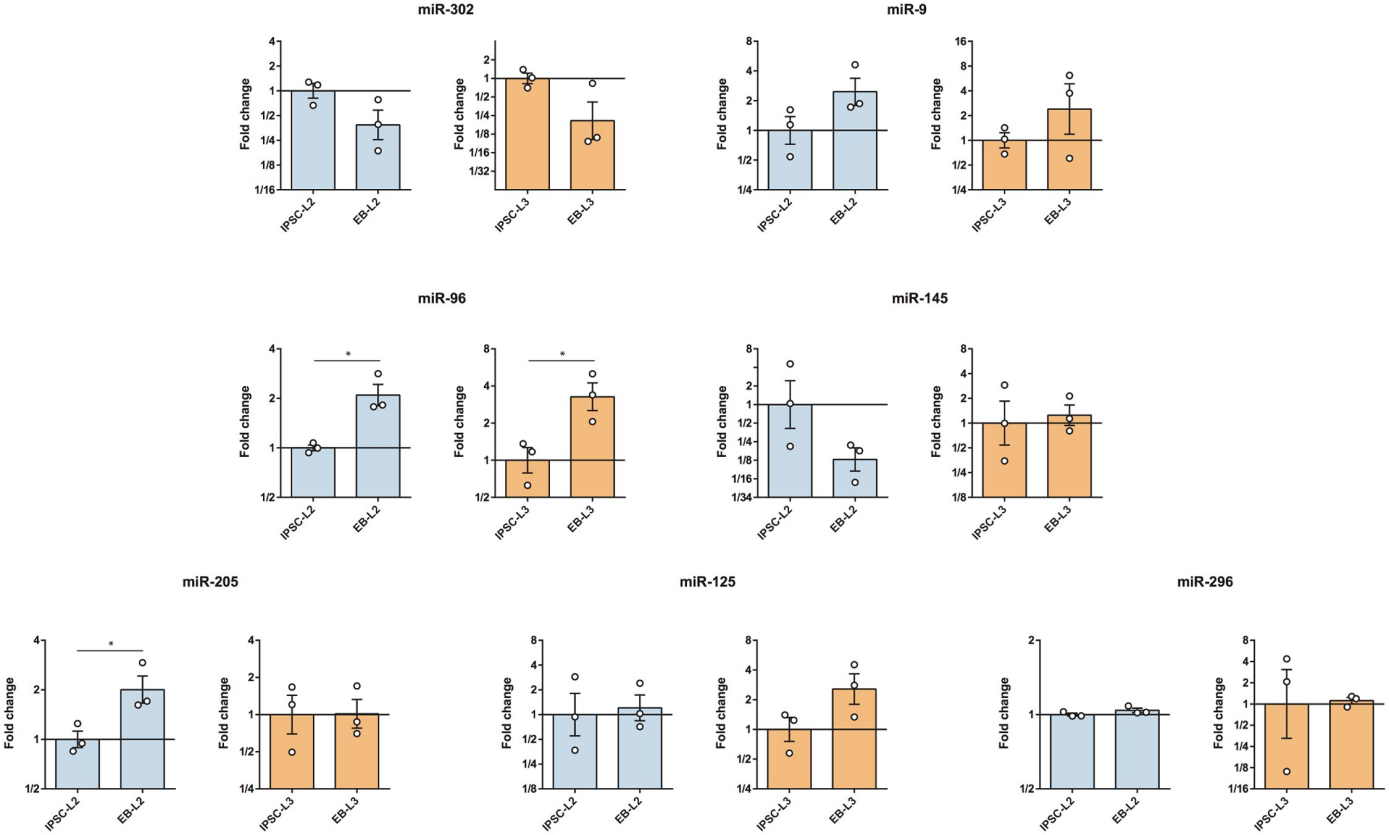
RT-qPCR analysis of 7 microRNAs in the iPSCs lines and the embryo bodies (EBs) derived from them, iPSC-L2 vs. EB-L2 and iPSC-L3 vs. EB-L3. Results are presented as means ± SEM (n = 3). EB-L2 data were relativized to iPSC-L2 data and EB-L3 data were relativized to iPSC-L3 data. *Statistically different (p<0.05).

The expression of 7 different microRNAs were evaluated in fibroblasts, iPSCs lines and EBs (Figs 4 and 5). We observed that the expression of the miR-302 family (miR-302a, miR-302b, miR-302c and miR-302d) was strongly induced after cell reprogramming (p<0.05). In addition to the miR-302, the miR-9 and the miR-96 were also up-regulated (p<0.05). In all cases, no differences were observed between the two iPSCs lines. After differentiation to EBs, the miR-96 was upregulated in the EBs respect to the iPSCs, and also the expression of miR-205 was induced but only in the EB-L2 (p<0.05). Finally, while miR-302 was downregulated in EBs, miR-9 was upregulated compared to iPSCs.

### microRNAs seed alignments

**Fig 6:**
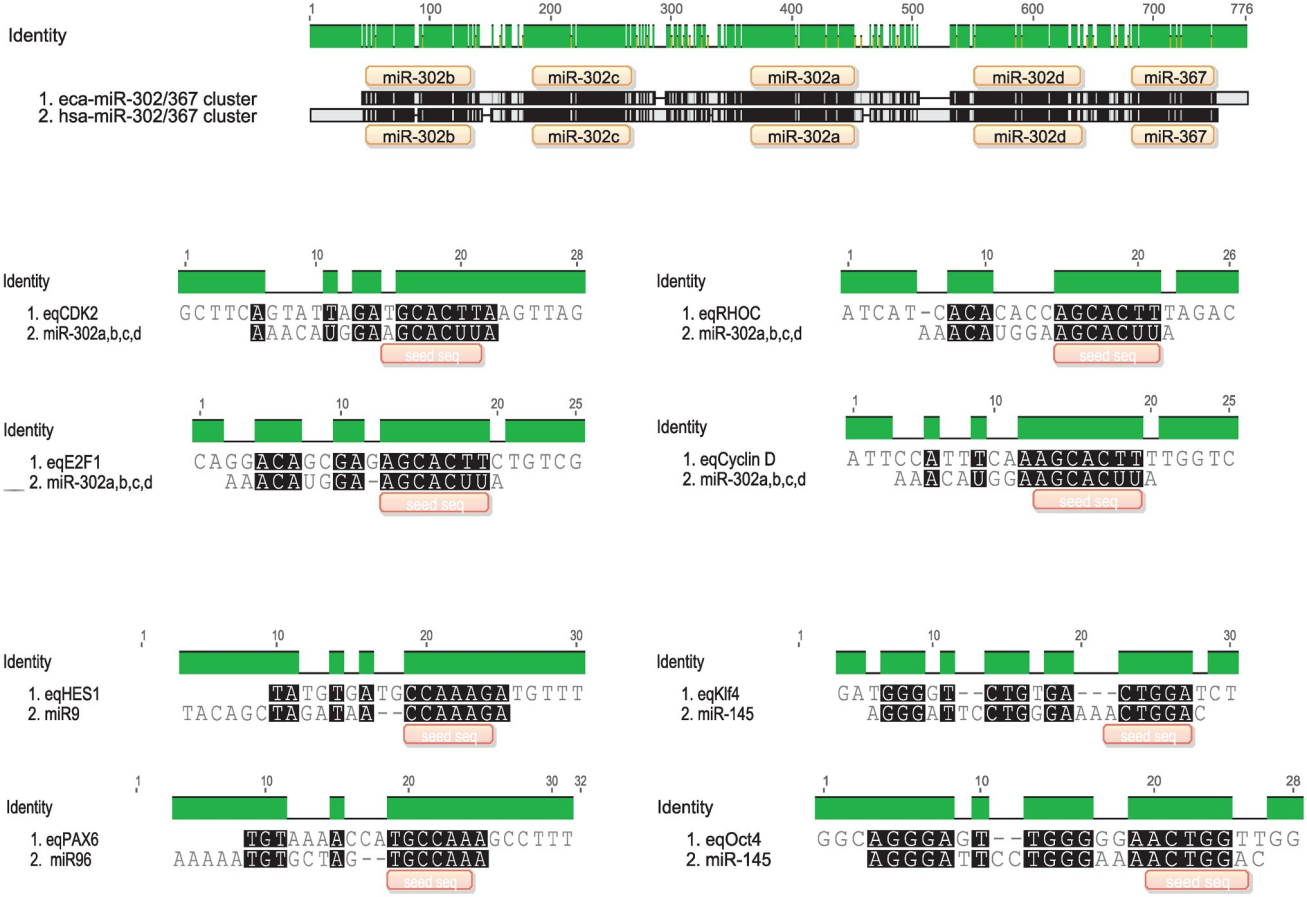
*In-silico* analysis of equine microRNAs targets. The green bars and the black zones represent the homology between sequences. a) Comparison between equine miR-302/367 cluster (eca-miR-302/367) and human miR-302/367 cluster (hsa-miR-302/367) in the genome. In orange are the different microRNAs positioned in the cluster. Below this alignment, the seed sequence of the miR-302 family is aligned to the 3‘UTR of 4 mRNAs in the equine (*CDK2*, *E2F1*, *RHOC* and *CYCLIN D*). b) Seed alignment of the differentiation-related microRNAs miR-9, miR-96 and miR-145, to the 3‘UTR of the equine mRNAs *HES1*, *PAX6* and *KLF4* and *OCT-4*, respectively.

We performed *in silico* analysis of some microRNAs evaluated in this paper. First, we compared the eca-miR-302/367 cluster with the hsa-miR-302/367 cluster and we determined a 75% homology between them (Fig 6a). Moreover, the seed region of the eca-miR-302 family resulted complementary to the 3‘UTR of horse cell cycle regulator genes *CDK2*, *CYCLIN D1* and *E2F1*, and to the 3‘UTR of the *RHOC* gene, which is involved in the epithelial-mesenchymal transition (Fig 6a). The miR-145 seed sequence was complementary to the 3‘UTR region of the Oct-4 and Klf-4 horse genes (Fig 6b). Respect to the miR-9 and miR-96, the seed sequence of these genes were complementary to HES1 and PAX-6 genes, as it was previously demonstrated in humans [27–29]. We also analyzed the target genes with the RNA22 software, confirming the complementarity evaluated by the seed sequence (S2 Fig).

## Discussion

In this report we generated two horse iPSCs lines by using the lentiviral vector STEMCCA containing the human reprogramming factors OSKM [32]. We cultured the iPSC for more than 25 passages without losing their pluripotent characteristics and proliferation capacity. In addition to their morphology, pluripotency was assessed by the expression of *OCT-4*, *NANOG* and *REX-1* by end point PCR and qPCR. Similarly as in equine embryos, equine iPSCs have demonstrated to widely express these markers [8, 9, 41–43]; however, only *REX1* has been associated exclusively with equine pluripotent cells [42], observing a significant expression of this gene in our iPSCs lines. Furthermore, we confirmed staining for OCT-4, SOX-2 and C-MYC by immunocytochemistry and also high alkaline phosphatase activity. Until now, no reliable equine ESC could be isolated in this species [1, 44, 45], which makes the pluripotent characterization of the equine iPSCs more challenging [1].

In addition to the identification of these pluripotent markers, both iPSCs lines could differentiate *in vitro* and form EBs. These EBs showed gene expression for markers of the three germ layers including *NESTIN*, *βIII-TUBULIN*, *AFP*, *VIMENTIN* and *BRACHYURY*, not observing expression of these genes in the original iPSCs. Moreover, we characterized the EBs by immunocytochemistry, observing positive staining for markers of the three embryonic germ layers GATA-4, *β*III-TUBULIN, VIMENTIN, SMA and NKX-2.5, despite protein analysis in this model is difficult to achieve due to a lack of reliable antibodies.

We also tried to generate equine iPSCs lines with different combinations of episomal vectors that have demonstrated their potential to efficiently reprogram human cells [38–40]. We could corroborate the incorporation of the plasmids by co-transfecting a reporter vector with GFP, but we could not reprogram equine fibroblasts by this technique. Besides human cells, episomal reprogramming was efficient in few species including only the mouse and the rat [12, 46], but was not efficient to generate pig iPSCs [47]. Apart from this report, no other one has been published in domestic species. The strongest hypothesis that might explain the inability to generate equine iPSCs with episomal vectors is that domestic iPSCs would require continuous expression of the transgenes to maintain pluripotency, as was previously observed [7, 48–50]. This hypothesis is also consistent with our cloning results with one of the iPSC lines. Whereas 2 viable foals were generated with the original fibroblasts, no blastocysts were obtained when the iPSCs were used as nuclear donors. The same result was previously published in the horse with a different iPSC line [35]. The continuous expression of the transgenes could be blocking specific cell determination during the first differentiation steps after morula formation.

In addition to the analysis of mRNAs we were also interested in the expression of microRNAs in horse iPSCs in order to expand our knowledge of the identity of pluripotent cells in this species. In human embryonic stem cells, specific microRNAs such as the miR-302 family are tightly connected with *OCT-4*, *SOX-2* and *NANOG* mRNAs in a relative expression level that is carefully balanced in order to maintain pluripotency [51, 52]. Conversely, it was reported that miR-145 represses the expression of *OCT-4*, *SOX-2* and *KLF4* [25] and it is down-regulated in iPSCs when compared with fibroblasts [14], thus controlling reprogramming and differentiation by targeting these stem factors. In our work, we observed similar results with the equine iPSCs lines. We also determined that the expression of the miR-302 family was induced in both iPSCs lines respect to the original fibroblasts. In addition, despite no differences were seen in the expression of miR-145 among the groups because of the data disparity, there is a tendency of miR-145 to be down-regulated in both horse iPSCs lines respect to the original fibroblasts, as was reported in human and pig iPSCs [53]. In consistence, the eca-miR-302/367 cluster conserves the homology and distribution in the genome similarly as in humans. Moreover, the miR-302 family seed sequence resulted complementary to *eqCyclin D1* 3‘UTR, *eqCDK2* 3‘UTR and *eqE2F1* 3‘UTR, important cell cycle regulators, and *eqRHOC*, a regulator of the mesenchymal-epithelial transition (MET). *In silico* alignments determined that the eca-miR-145 seed sequence was complementary to the 3‘UTR of pluripotent genes including *eqKLF4* 3‘UTR and *eqOCT-4* 3‘UTR. These determinations complemented our experimental results and let us speculate that there are similar regulations of miR-302 family and miR-145 as in humans [25, 26, 51, 54].

Other differentiation-related microRNAs as the miR-9 and miR-96 are involved in neural specification. miR-9 promotes neural lineage differentiation by inhibiting neural stem cells proliferation [55–57], and miR-96 is over-expressed in human pluripotent cells, and it is also involved in PAX6 repression, inhibiting neural induction [28, 58]. Here we observed that miR-9 and miR-96 were also over-expressed in L2 and L3 iPSCs respect to the original fibroblasts and that miR-96 significantly enhanced its expression in the differentiated cells to EBs respect to the iPSCs. Whether this regulation is related to neural specification was not determined experimentally, but the seed sequence reported for the hsa-miR-96 [28] that is the same as the eca-miR-96 is complementary to the 3´UTR of the eqPAX6 gene. Moreover, miR-9 has demonstrated to bind the 3‘UTR of the HES1 mRNA in the developing brain of the mouse, regulating the proliferation and differentiation of neural stem cells [55]. *In silico* alignments demonstrated that the eca-miR-9 may be able to bind the 3′-UTR of eqHes1 mRNA by its seed sequence, thus regulating the expression of this gene. With these results we consider that both miR-96 and miR-9 may also be involved in neural specification in the horse.

The other microRNAs evaluated after in vitro differentiation to EBs, did not show statistical differences respect to the iPSCs lines, but miR-302 tends to be downregulated and miR-9 tends to be upregulated in the EBs. Because the EBs have a mix of heterogenous population of cells differentiated to the three germ layers, it is possible that it could be some compensation in the quantification of the expression of the microRNAs.

## Conclusion

In summary, we generated and characterized two horse iPSC cell lines derived from embryonic fibroblasts by lentiviral infection of the reprogramming factors, but we were not able to reprogram the same fibroblasts by using episomal vectors. Moreover, several results led us to think that the equine microRNAs evaluated in our work are highly conserved in sequence and function respect to the human species. Now, it is necessary to generate directed differentiations to derivatives of the three germ layers in order to strengthen our results. This is the first report to evaluate the expression and possible targets of microRNAs in domestic animals pluripotent cells.

## Supporting information

S1 Fig Immunostainig of horse embryos at the blastocyst stage. Positive staining for the pluripotent markers OCT-4, SOX-2 and c-MYC in equine embryos. In blue nucleous are stained with DAPI.
(TIF)

S2 Fig RNA22 output results. Complementarity analysis of equine microRNAs with target transcripts.
(TIFF)

S1 Table List of primers used in this study. (DOCX)

S2 Table List of antibodies used in these paper. (DOCX)

S3 Table Episomal reprogramming. Details about protocols used for cell reprogramming using episomal vectors.
(DOCX)
