# Supplementary figures and images for: MicroRNA characterization in equine induced pluripotent stem cells
Source: PLoS One. 2018 Dec 3;13(12):e0207074. doi: 10.1371/journal.pone.0207074 (PMC6277106; doi:10.1371/journal.pone.0207074)

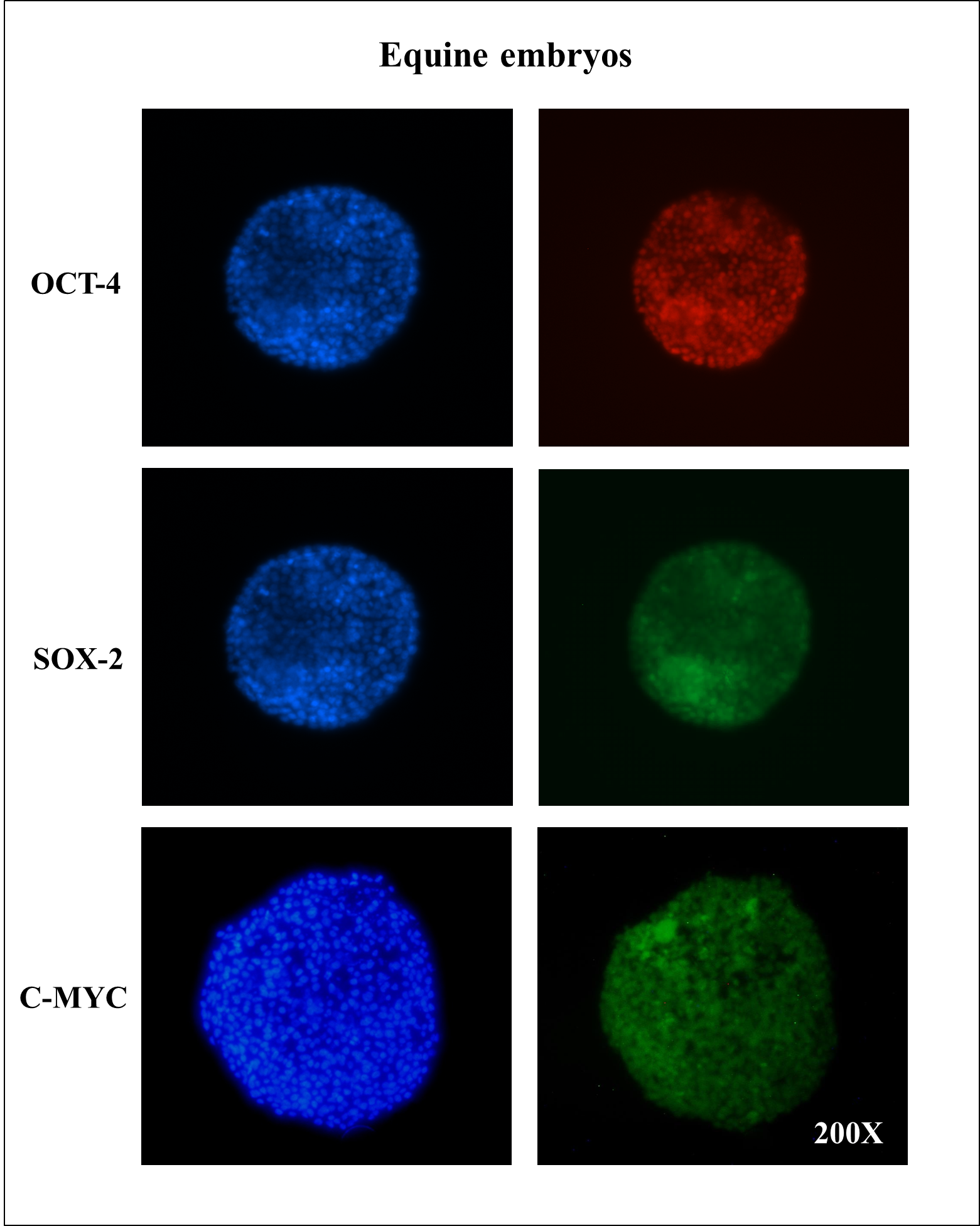

Supplement: S1 Fig — Positive staining for the pluripotent markers OCT-4, SOX-2 and c-MYC in equine embryos. In blue nucleous are stained with DAPI. (TIF) [file pone.0207074.s001.tif]
