# Supplementary material for: MicroRNA characterization in equine induced pluripotent stem cells
Source: PLoS One. 2018 Dec 3;13(12):e0207074. doi: 10.1371/journal.pone.0207074 (PMC6277106; doi:10.1371/journal.pone.0207074)
Supplement: S1 Table — (DOCX) [file pone.0207074.s003.docx]

| **Protocols** | **Total DNA (ug)** | **Plasmids used (addgene code)** | **NEON conditions** |
| --- | --- | --- | --- |
| **1** | 3 | 27077, 27078, 27080 | 1600 V, 3 pulses 10 ms |
| **2** | 6 | 27077, 27078, 27080 | 1600 V, 3 pulses 10 ms |
| **3** | 3 | 27077, 27078, 27080 | 1800 V, 3 pulses 10 ms |
| **4** | 4 | 27077, 27078, 27080, 20924 | 1600 V, 3 pulses 10 ms |
| **5** | 4 | 27077, 27078, 27080, 20924 | 1800 V, 3 pulses 10 ms |
| **6** | 1, 6 | 27077, 27078, 27082, 37624 | 1600 V, 3 pulses 10 ms |
| **7** | 3 | 27077, 27078, 27082, 37624 | 1600 V, 3 pulses 10 ms |
| **8** | 3 | 27077, 27078, 27082, 37624 | 1400 V, 2 pulses 10 ms |
|  |  |  | **Liposomes** |
| **9** | 1, 6 | 27077, 27078, 27082, 37624 | 1:3 DNA-liposome |
| **10** | 3,2 | 27077, 27078, 27082, 37624 | 1:3 DNA-liposome |

Supplementary table S1
