# Supplementary material for: MicroRNA characterization in equine induced pluripotent stem cells
Source: PLoS One. 2018 Dec 3;13(12):e0207074. doi: 10.1371/journal.pone.0207074 (PMC6277106; doi:10.1371/journal.pone.0207074)
Supplement: S2 Table — (DOCX) [file pone.0207074.s004.docx]

| **Genes** | **Primer Sequences** |
| --- | --- |
| Oct4 | F: 5´-GAGGCTCTGCAGCTCAGTTT-3´ |
|  | R: 5´-CTCCAGGTTGCCTCTCACTC-3´ |
| Nanog | F: 5´-ATACCTCAGCCTCCAGCAGA-3´ |
|  | R: 5´-AGCCCCGGGTAGTCTGTAGT-3´ |
| Rex1 | F: 5´-CTTGAAGAAGCCCATTATCC-3´ |
|  | R: 5´-GCCACACTCAGGTAGATG-3´ |
| Vimentin | F: 5´-TCGCCAACTACATCGACCAA-3´ |
|  | R: 5´-CTTCTTTGGCACGTCTTGACC-3´ |
| Nestin | F: 5´-TGGAGCAGAGGTTGGAGGG-3´ |
|  | R: 5´-TTGCTTTCTGTCCTGAGCCC-3´ |
| BIII-Tubulin | F: 5´-AGCGCATCAGCGTCTACTAC-3´ |
|  | R: 5´-GTGCGGAAGGAGTGTGAGAA-3´ |
| AFP | F: 5´-GCAACTATGAAGTGGGTGGT-3´ |
|  | R: 5´-GTTTACTGGCACGCAAGAAGG-3´ |
| RPL7 | F: 5´-CAAGGAGTATAGGCAGATG-3´ |
|  | R: 5´-CGAGCAATCAATGAGTTATC-3´ |
| **miRNAs** |  |
| miRNAs RV | R:5´-ATCCAGTGCAGGGTCCGAGG-3´ |
| miR-9 5p | F: 5´-GCGCGGTCTTTGGTTATCTAG-3´ |
| miR-96 5p | F: 5´-GCGGCGGTTTGGCACTAGCAC-3´ |
| miR-125 b | F: 5´-GCGGCGGTCCCTGAGACCCTAAC-3´ |
| miR-145 | F: 5´-GCGGCGGGTCCAGTTTTCCCAG-3´ |
| miR-296 | F: 5´-GCGGCGGAGGGCCCCCCCTCAATC-3´ |
| miR-205 | F: 5´-GCGGCGGTCCTTCATTCCACCG-3´ |
| miR-302 family | F: 5´-GCGGCGGTAAGTGCTTCCATG-3´ |
| Rnu6b | F: 5´-CGCAAGGATGACACGCAAATTC-3´ |
|  | R: 5´-CCAGTGCAGGGGTCCGAGGT-3´ |

| **miRNAs** | **Stem loop primers** |
| --- | --- |
| miR-9 5p | GTCGTATCCAGTGCAGGGTCCGAGGTATTCGCACTGGATACGACTCATAC |
| miR-96 5p | GTCGTATCCAGTGCAGGGTCCGAGGTATTCGCACTGGATACGACAGCAAA |
| miR-125 b | GTCGTATCCAGTGCAGGGTCCGAGGTATTCGCACTGGATACGACTCACAA |
| miR-145 | GTCTCCTCTGGTGCAGGGTCCGAGGTATTCGCACCAGAGGAGACAGGGAT |
| miR-296 | GTCTCCTCTGGTGCAGGGTCCGAGGTATTCGCACCAGAGGAGACACAGGA |
| miR-205 | GTCGTATCCAGTGCAGGGTCCGAGGTATTCGCACTGGATACGACCAGACT |
| miR-302a | GTCTCCTCTGGTGCAGGGTCCGAGGTATTCGCACCAGAGGAGACTCACCA |
| miR-302b | GTCTCCTCTGGTGCAGGGTCCGAGGTATTCGCACCAGAGGAGACCTACTA |
| miR-302c | GTCTCCTCTGGTGCAGGGTCCGAGGTATTCGCACCAGAGGAGACCCACTG |
| miR-302d | GTCTCCTCTGGTGCAGGGTCCGAGGTATTCGCACCAGAGGAGACACACTC |
| Rnu6b | GTCGTATCCAGTGCAGGGTCCGAGGTATTCGCACTGGATACGACAAAATATGGAAC |
