# Supplementary material for: MicroRNA characterization in equine induced pluripotent stem cells
Source: PLoS One. 2018 Dec 3;13(12):e0207074. doi: 10.1371/journal.pone.0207074 (PMC6277106; doi:10.1371/journal.pone.0207074)
Supplement: S3 Table — Details about protocols used for cell reprogramming using episomal vectors. (DOCX) [file pone.0207074.s005.docx]

| **Antibody** | **Catalogue/Supplier** | **Species** | **Dilution** |
| --- | --- | --- | --- |
| OCT4 | sc 5279/Santa ruz Biotechnology | Mouse | 1/200 |
| SOX 2 | 35795S/Cell Signaling | Rabbit | 1/200 |
| C-MYC | 5605P/Cell signaling | Rabbit | 1/200 |
| GATA 4 | sc 25310/Santa Cruz Biotechnology | Mouse | 1/200 |
| BIII-TUBULIN | AB78078/Biolegend | Mouse | 1/200 |
| VIMENTIN | NCL-VIM-V9/Leica Biosystems | Mouse | 1/200 |
| SMA | A5228/Sigma | Mouse | 1/200 |
| NKX 2.5 | sc 14033/Santa Cruz Biotechnology | Rabbit | 1/200 |
| Anti-mouse Alexa Fluor 555 | A21424/ThermoFisher | Goat | 1/1000 |
| Anti-rabbit Alexa Fluor 555 | A31572/ ThermoFisher | Donkey | 1/1000 |
| Anti-mouse Alexa Fluor 488 | A11029/ ThermoFisher | Goat | 1/1000 |
| Anti-rabbit Alexa Fluor 488 | A11034/ ThermoFisher | Goat | 1/1000 |
